# The Registration Fee System

**Published:** 1886-10-30

**Authors:** 

**Affiliations:** Lord-Lieutenant of Warwickshire


### The Registration Fee System.

By The Right Hon. LORD LEIGH,

Lord-Lieutenant of Warwickshire.

The Provident Principle.

I have for many years entertained a very strong opinion that it is desirable in all hospitals and similar institutions, to carry out the provident principle, and I hope to live to see the day when not only at Warneford, but at every hospital in England, some small sum may be paid as an entrance-fee. I have several reasons for entertaining this hope. In the first place, it is far better for individuals who receive relief from these institutions to pay a fee, however small, as it gives them a direct interest in the work done, and thereby promotes the efficiency of the institution. I have for many years had the honour of being president of a hospital—the Queen's at Birmingham—which is conducted on the principle I am now advocating. It is true that hospital does not give out tickets, but that is no reason why Warneford, or any other ticket hospital, should not be conducted in the same way. It is said Warneford is a different hospital from the Queen's at Birmingham, and that the system would not answer in Leamington. Will anyone explain why? As regards efficiency, it is easy to show that the hospital would be greatly benefited by the pay system.

The Fee System at Work.

The plan was introduced in Birmingham in 1876—ten years ago. I have a letter from the secretary, of recent date, giving details as to its working during that period. The letter states that, up to the present time, the system has worked most satisfactorily, and the sums realised from fees since 1876 have been as follow:—1876, £497 17*s.*; 1877, £524 17*s.*; 1878, £539 10*s.*; 1879, £527 2*s.*; 1880, £517 7*s.*; 1881, £536 11*s.*; 1882, £579 3*s.*; 1883, £676 8*s.*; 1884, £811 8*s.*; 1885, £852 17*s.* These figures show an almost uninterrupted rate of increase for the decade, and the amount for 1885 is nearly double that for 1876. The system is worked in the following manner:—The patients attend at the hospital between 9 and 11 a.m., when certain questions are put to each of them. If the registrar is satisfied that the applicant is in a position to pay a fee of 1*s.*, then the fee has to be paid; but if from any cause he is unable, the fee is remitted. These remittances amount to nearly 10 per cent.

Lord Leigh's Proposal.

What I would propose is, that a registration-fee of 1*s.* be paid in every instance. Of course, in any case of destitution it should be in the power of the medical superintendent, or officer in charge, to remit the fee. In ordinary cases where a first entrance-fee has been paid, a second should not be asked for at the expiration of a four weeks' ticket. If a renewal of the ticket be required, it should be granted, at any rate *once*, without further fee. It is sometimes said that the hospital, being a charity, ought not to take payment from patients; but I would like to ask whether an entrance-fee of 1*s.* for one and sometimes two months' medical assistance can reasonably be designated *payment?* I can hardly imagine even a chemist's errand-boy who would not consider his services worth more than 1*s.* for four weeks. This being the case, the question of charity may at once be dispensed with, and the only point that remains is the question of expediency.

The Warneford Hospital.

We are frequently told that it is very up-hill work to manage the Warneford Hospital efficiently owing to the want of funds. Unfortunately, during the last year or so, we have lost many good supporters by death, and it is undoubtedly a fact that we have to struggle very hard to meet necessary expenditure. Now, I find that in 1885 there were 1,728 out-patients at the hospital who enjoyed its benefits by virtue of tickets; and if, following out what has been done at the Queen's Hospital, Birmingham, they had each paid a registration-fee of 1*s.*, it would have amounted to £86 8*s.* Taking from this the ten per cent. remittances, there would have been left £78 16*s.*, which could have been applied to carrying on the hospital, and it would still have been to all intents and purposes a charitable institution. Nobody would have been injured, and the hospital would have been richer by nearly eighty pounds.

Does it promote Begging?

Again, it has been stated that the system will give rise to great imposition, and that the people who get the tickets will go about stating they have not the 1*s.*, and will beg from one place to another, and, perhaps, instead of 1*s.* they might get 20*s.* I cannot for the life of me see why this objection cannot be met. In any case, it is a matter of detail which can surely be easily dealt with, if the provident principle is adopted. It would be the simplest thing in the world for those who gave the ticket to the patient to sign their names on it, and to state they had paid the regulation fee. That this difficulty is purely imaginary has been fully demonstrated at Birmingham by ten years' experience.

A Strong Opinion. I feel very strongly indeed on the subject, and I hope that the principle will ultimately be adopted, not only at Warneford, but at every other hospital in the kingdom. If this were done in the hospitals generally, it would tend to promote their efficiency, and give an interest to the poor patients themselves, which would be of the greatest possible benefit to the country. Holding these views, I look forward with hope to the time when all ordinary out-patients, admitted by ticket, will have the privilege of contributing, according to their ability, to the maintenance and efficiency of these noble institutions.

